# A Bispecific Antibody Promotes Aggregation of Ricin Toxin on Cell Surfaces and Alters Dynamics of Toxin Internalization and Trafficking

**DOI:** 10.1371/journal.pone.0156893

**Published:** 2016-06-14

**Authors:** Cristina Herrera, Tove Irene Klokk, Richard Cole, Kirsten Sandvig, Nicholas J. Mantis

**Affiliations:** 1 Division of Infectious Disease, Wadsworth Center, New York State Department of Health, Albany, New York, United States of America; 2 Department of Biomedical Sciences, University at Albany School of Public Health, Albany, New York, United States of America; 3 Department of Molecular Cell Biology and Centre for Cancer Biomedicine, Institute for Cancer Research, The Norwegian Radium Hospital, Oslo University Hospital, Montebello, Oslo, Norway; 4 Department of Biosciences, University of Oslo, Oslo, Norway; 5 Division of Translational Medicine, Wadsworth Center, New York State Department of Health, Albany, New York, United States of America; Institut Curie, FRANCE

## Abstract

JJX12 is an engineered bispecific antibody against ricin, a member of the medically important A-B family of toxins that exploits retrograde transport as means to gain entry into the cytosol of target cells. JJX12 consists of RTA-D10, a camelid single variable domain (V_H_H) antibody directed against an epitope on ricin’s enzymatic subunit (RTA), linked via a 15-mer peptide to RTB-B7, a V_H_H against ricin’s bivalent galactose binding subunit (RTB). We previously reported that JJX12, but not an equimolar mixture of RTA-D10 and RTB-B7 monomers, was able to passively protect mice against a lethal dose ricin challenge, demonstrating that physically linking RTB-B7 and RTA-D10 is critical for toxin-neutralizing activity *in vivo*. We also reported that JJX12 promotes aggregation of ricin in solution, presumably through the formation of intermolecular crosslinking. In the current study, we now present evidence that JJX12 affects the dynamics of ricin uptake and trafficking in human epithelial cells. Confocal microscopy, as well as live cell imaging coupled with endocytosis pathway-specific inhibitors, revealed that JJX12-toxin complexes are formed on the surfaces of mammalian cells and internalized via a pathway sensitive to amiloride, a known inhibitor of macropinocytosis. Moreover, in the presence of JJX12, retrograde transport of ricin to the trans-Golgi network was significantly reduced, while accumulation of the toxin in late endosomes was significantly enhanced. In summary, we propose that JJX12, by virtue of its ability to crosslink ricin toxin, alters the route of toxin uptake and trafficking within cells.

## Introduction

Ricin, a member of the A-B family of bacterial and plant proteins toxins, is classified by the Centers for Disease Control and Prevention (CDC) as a potential biothreat agent [1, 2]. Ricin’s enzymatic subunit (RTA) is an RNA *N*-glycosidase that inactivates eukaryotic ribosomes by catalyzing the hydrolysis of a conserved adenine residue within the so-called sarcin/ricin loop (SRL) of 28S rRNA [3, 4]. Ricin’s B subunit (RTB) is a galactose- and N-acetylgalactosamine (Gal/GalNAc)-specific lectin that promotes ricin clathrin-dependent and independent endocytosis into mammalian cells, including epithelial cells that line the respiratory tract [5, 6]. Following uptake, RTB also mediates the retrograde transport of ricin to the trans-Golgi network (TGN) and endoplasmic reticulum (ER). Once within the ER, the single disulfide bond that links RTA to RTB is reduced by protein disulfide isomerase and RTA is then retro-translocated (dislocated) into the cell cytoplasm where it promotes ribosome inactivation and cell death [7, 8].

Although we and others have produced a number of chimeric and humanized monoclonal antibodies (mAbs) against ricin toxin with promising therapeutic value [9–12], much remains to be understood regarding optimal strategies for inactivating ricin *in vivo*. Of particular interest are antitoxin agents based on toxin-specific camelid heavy-chain only VH domains (V_H_Hs) antibodies [13]. V_H_Hs have been identified against botulinum neurotoxin (BoNT) [14], *Clostridium difficile* toxins TcdA and TcdB [15–17], Shiga toxins [18], and anthrax toxin [19]. While monomeric V_H_Hs generally have little toxin-neutralizing activity *in vivo*, bispecific (heterodimeric) V_H_H constructs consisting of two different V_H_Hs joined via a flexible peptide linker have proven to be remarkably effective at affording passive protection against extraordinarily high dose toxin challenges [14, 20] or even, in the case of anthrax toxin, a spore challenge [19]. The prophylactic and therapeutic potential of these so-called VHH-based neutralizing agents (VNAs) is even more remarkable considering that they have been successfully engineered and administered to mice and piglets via a non-replicating adenovirus vector [14, 21, 22].

We recently produced and characterized a collection of ricin toxin-specific V_H_Hs from immunized alpacas [23]. For the sake of the current study, two specific V_H_Hs are of interest: RTA-D10 and RTB-B7. RTA-D10 recognizes an immunodominant epitope on RTA, as was revealed by the X-ray crystal structure of RTA-D10 in complex with RTA [24]. RTA-D10 neutralizes ricin *in vitro* with an IC_50_ of ~25 nM, thereby ranking it as having moderate toxin-neutralizing activity (TNA). RTB-B7 is proposed to recognize an epitope situated within near one of RTB’s two galactose binding sites and has an IC_50_ of ~1.5 nM, is one of the most potent neutralizing antibodies identified to date [25]. However, neither RTA-D10 nor RTB-B7 is able to fully neutralize ricin toxin *in vivo*, even when administered concomitantly with ricin [23, 25]. Similarly, a mixture of RTA-D10 and RTB-B7 also failed to neutralize ricin in a mouse model, although that dynamic changed considerably when the two V_H_H constructs were joined by a flexible 15-mer peptide linker and expressed as a bispecific antibody known as JJX12 [23]. Specifically, we demonstrated that JJX12 was able to fully protect mice from a 10 x LD_50_ ricin challenge at antibody:ricin ratios as low as 4:1, whereas a mixture of RTA-D10 and RTB-B7 monomers had no appreciable protective activity [23]

The fact that JJX12, but not an equimolar mixture of RTA-D10 and RTB-B7 monomers, was able to passively protect mice against a lethal dose ricin challenge, demonstrates that physically linking RTB-B7 and RTA-D10 is critical for toxin-neutralizing activity *in vivo*. The bispecific nature of JJX12 is also important because neither JNA6, a homodimer of RTB-B7, nor JNA3, a homodimer of RTA-D10 was able to neutralize ricin *in vivo* [26]. Because the linker (GGGGS)_3_ that joins RTB-B7 and RTA-D10 in JJX12 is theoretically too short to permit RTB-B7 and RTA-D10 to simultaneously bind the same ricin molecule, we postulated that JJX12 must neutralize ricin through the formation of inter- rather than intra-molecular toxin binding. Consistent with this model, we demonstrated using analytical ultracentrifugation (AUC) that JJX12 (but not JNA6 nor RTB-B7) promotes formation of high molecular weight toxin-antibody complexes in solution [25, 26]. Other bispecific antibodies in which RTB-B7 was linked to an RTA-specific V_H_H also displayed the capacity to form high molecular weight toxin-antibody complexes in solution [26].

It has been recognized for more than three decades that factors that influence the valency and/or size of ricin can affect the route by which ricin gains entry into host cells, as well as the efficiency of toxin retrograde transport to the TGN [27]. Therefore, the goal of the current study was to test the hypothesis that JJX12, by virtue of its ability to crosslink ricin, alters the mechanism by which the toxin is internalized and trafficked within mammalian cells.

## Materials and Methods

### Chemicals, biological reagents and cell lines

Ricin toxin (*Ricinus communis* agglutinin II), biotinylated ricin, and ricin-FITC (fluorescein isothiocyanate) were purchased from Vector Laboratories (Burlingame, CA). Ricin was dialyzed against PBS at 4°C in 10,000 molecular weight cutoff Slide-A-Lyzer dialysis cassettes (Pierce, Rockford, IL) prior to use. D-(+)- lactose was obtained from J.T. Baker (Center Valley, PA) and asialofetuin (ASF) from Sigma-Aldrich (St. Louis, MO). Goat serum was purchased from Gibco-Invitrogen (Carlsbad, CA). Anti-E-tag horseradish peroxidase (HRP) conjugated mAb was purchased from Bethyl Laboratories, Inc. (Montgomery, TX) and streptavidin HRP conjugated was purchased from Thermo Fisher Scientific (Waltham, MA). Unless noted otherwise, all other chemicals were obtained from Sigma-Aldrich. Cell culture media were obtained from the tissue culture core facility at the Wadsworth Center. THP-1 cells were obtained from the American Type Culture Collection (ATCC; Manassas, VA) and were grown in RPMI supplemented with 10% fetal bovine serum (FBS). The human lung epithelial cell line A549 was also purchased from ATCC and was grown in DMEM with 10% FBS. Cells were maintained in incubators set at 37°C with 5% CO_2_ atmosphere. Dynasore and the amiloride analog 5-(N-Ethyl-N-isopropyl; EIPA) were purchased from Sigma Aldrich; latrunculin A (LatA) was obtained from Thermo Fisher Scientific. CellLight-RFP, BacMam 2.0 was used to label the trans-Golgi network (TGN), late endosomes, or lysosomes (Thermo Fisher Scientific). JNA6 and JJX12 were directly labeled using Alexa Fluor-633 and -647 Protein Labeling Kits following manufacturer’s protocol (Thermo Fisher Scientific).

### VHH expression and purification

RTB-B7, JNA6, and JJX12 (Table 1) were purified using a nickel affinity column (Thermo Fisher Scientific) to the vector-encoded hexahistidine, as previously reported [26]. RTB-B7, JNA6, and JJX12 each carry a carboxyl terminal E-tag epitope, which can be used for detection purposes with anti-E-tag secondary antibody. Purity and concentrations of the antibodies was determined by SDS-PAGE with comparisons to internal standards.

**Table 1:** Engineered V_H_H antibodies used in this study.

V_H_H	Constituents ^a^	References
JJX12	RTB-B7 + RTA-D10	[23, 26]
JNA6	RTB-B7 + RTB-B7	[26]
RTB-B7	n.a.	[23, 25]

^*a*^ Corresponding neutralizing monomers for homodimer and heterodimer;

n.a, not applicable.

### Ricin binding assay using flow cytometry

THP-1 cells were collected by centrifugation (5 min at 400 x *g*) and the resulting cell pellets were suspended to 5x10^6^ cells per ml, as described [25]. The cells were seeded (200 μl/well) into clear U-bottom 96-well plates (BD Bioscience; San Jose, CA). Ricin-FITC was mixed with antibodies and incubated in the dark for 30 min on ice. Cells were washed with PHEM buffer [60 mM piperazine-*N*,*N′*-bis(2-ethanesulfonic acid) (PIPES), 25 mM HEPES, 10 mM EGTA, 2 mM MgCl2, pH 6.9] to remove unbound ricin:antibody complexes, then incubated with 0.1 M lactose to remove surface bound toxin. The cells were re-suspended in sorting buffer [1x PBS, 25 mM HEPES, 1 mM EDTA] prior to analysis using a FACS Calibur flow cytometer (BD Bioscience). A minimum of 10,000 events was analyzed per sample.

### Ricin competition assays with lactose

For lactose competition ELISAs, Nunc Immuno MicroWell 96 well plates (Thermo Fisher Scientific) were coated overnight with ASF (0.4 μg/well) in PBS (pH 7.4). The following day the plates were blocked with PBS (pH 7.4) containing 0.05% Tween-20 (PBS-T) and supplemented with 2% goat serum. Ricin or biotinylated ricin was applied to microtiter plates for 1 h at 25°C and then washed and treated with 2-fold dilutions (starting at 0.2 M) of lactose. Plates were probed with HRP-anti-E-tag (1:10,000) to detect VHHs or streptavidin-HRP (1:500) to detect biotinylated ricin, and developed with TMB [3,3′,5,5′-Tetramethylbenzidine] (KPL; Gaithersburg, MD). The reaction was stopped with 1 M phosphoric acid and absorbance was read at 450 nm using the VersaMax Microplate Reader with Softmax Pro 5.2 software (Molecular Devices; Sunnyvale, CA).

### Ricin sulfation assay to measure transport to TGN

Ricin-sulf-1 (RS1) was produced and purified as described [28, 29]. HeLa cells were washed with sulfate-free HEPES-buffered medium, followed by incubation with 0.2 mCi/ml Na_2_^35^SO_4_ in sulfate-free HEPES-buffered medium for 3 h at 37°C. RS1 was incubated with antibodies for 30 min at RT before addition to the cells and incubated for an additional 2 h. Medium was removed and cells washed twice with 0.1 M lactose in HEPES-buffered medium and once in cold PBS on ice before addition of 400 μl lysis buffer [0.1 M NaCl, 10 mM Na_2_HPO_4_, 1 mM EDTA, 1% Triton X-100, 60 mM octyl glycopyranoside] supplemented with complete protease inhibitors (Roche Diagnostics; Mannheim, Germany). Lysate was cleared by centrifugation (8,000 rpm for 10 min at 4°C) and 300 μl of the supernatant was mixed with 5% trichloro acetic acid (TCA) followed by centrifugation (14,000 rpm for 10 min at 4°C). After washing the pellet in ice-cold PBS, it was dissolved in 2x sample buffer and separated by SDS-PAGE under reducing conditions, followed by blotting onto a PVDF membrane (Immobilon-P, Millipore; Billerica, MA). The bands were detected by autoradiography using a PharosFX scanner and quantified using Quantity One^®^ 1-D Analysis Software (BioRad Laboratories Inc.; Hercules, CA). The total amount of sulfated proteins was determined by TCA-precipitation of the remaining lysates.

For the purpose of quantification of ricin internalization after the sulfation assay, the resulting PVDF membrane was re-wet in PBS-T (PBS with 0.01% Tween-20) and then probed overnight at 4°C with polyclonal anti-RTA antibody (Abcam, Cambridge, MA) in 5% BSA in PBS-T. The membrane was then repeatedly washed with PBS-T and probed with HRP-conjugated secondary antibody (Jackson Immunoresearch) that had been diluted in 1% BSA in PBS-T. The membrane were developed using ECL Western blotting detection reagent (GE Healthcare, Buckinghamshire, UK) and quantified using Quantity One^®^ 1-D Analysis Software (BioRad, Oslo, Norway). The densitometry signals in presence of antibodies were normalized to the signal for RS1 alone, which was set to 100%.

### Cell surface binding of ^125^I-ricin

The IODO-GEN Iodination Reagent was used for ^125^I-ricin labeling according to the manufacturer’s protocol (Thermo Fisher Scientific), using Na^125^I from PerkinElmer (Waltham, MA). The yield of iodine incorporation was approximately 50,000 cpm/ng of ^125^I-ricin. For analysis of ricin binding, antibodies were incubated with ^125^I-ricin in HEPES-buffered medium for 25 min at RT and 5 min on ice, before the mix was added to HeLa cells seeded in 24-well plates at a density of 5 x10^4^ cells per well. Cells had been incubated on ice for 20 min and washed with cold HEPES-buffered medium. After incubation with ^125^I-ricin-antibodies for 30 min on ice, cells were washed with cold PBS or 0.1 M lactose in HEPES-buffered medium, followed by lysis in 0.1 M KOH. The amount of cell-associated ^125^I-ricin was measured using a gamma counter (PerkinElmer).

### Live cell imaging and co-localization of ricin with organelles

A549 cells were seeded on MatTek dishes (3.5 x10^5^ cells total) (MatTek Corporation; Ashland, MA). Cells were incubated for 8 h and then transiently transfected with RFP CellLight constructs expressing RFP-tagged reporters for the TGN, late endosome, and lysosomes (BacMam 2.0 technology, Thermo Fischer Scientific) using 40 particles per cell. The following day cells were treated with ricin-FITC, with or without antibody addition, for 20 min at 37°C before being subject live cell imaging. Images were collected with the Nikon Ti inverted scope equipped for fluorescent imaging using 60x objective (1.4 NA) (Nikon, Inc.; Melville, NY). Images were collected in real time over 60 min using perfect focus. Live cell images were deconcolved using AutoQuant (Media Cybernetics; Rockville, MD) calculating the spherical aberration and refractive index for each image using 20–40 iterations. Analysis was done with FIJI is just ImageJ 1.50e [30]. 3D volume of each image was thresholded using the Otsu setting for both ricin-FITC and RFP channels (RFP-lysosomes thresholded using Yen), deleted out of focus slices for each 3D stack, and the region of interest (ROI) was selected using ImageJ’s freehand tool. Frequency of ricin co-localization with each organelle was determined using Mander’s coefficients calculated in ImageJ using Coloc2 plug in. Total ricin internalized was normalized for each individual cell, using the Max function in Microsoft excel (value/Max(range of time points)).

### Confocal microscopy and uptake inhibitors

A549 cells were seeded on MatTek dishes at a concentration of 3.5 x10^5^ cells (MatTek Corporation). Cells were then treated with each corresponding inhibitor (LatA, EIPA, or dynasore) dissolved in DMEM, for 30 min at 37°C. For experiments involving dynasore, cells were maintained in serum-free DMEM medium. After the initial 30 min incubation, the medium was removed and replenished with DMEM containing inhibitors plus ricin-FITC, ricin-FITC mixed with JJX12 or JNA6. The cells were incubated for 20 min, washed to remove unbound ricin or ricin:antibody complexes and then incubated with DMEM medium in the presence of inhibitor for an additional 30 min at 37°C. Finally, cells were washed with PHEM buffer, fixed with 2% glutaraldehyde, treated with sodium borohydride (NaBH_4_), and then washed prior to imaging. For actin staining, cells were incubated with phalloidin conjugated to Alexa-546. Fixed cells were imaged with the Leica TCS SP5 AOBS (acousto-optical beam splitter) confocal microscope with multiphoton laser and 63x objective (1.4 NA) (Leica Microsystems, Inc.; Buffalo Grove, IL). The system was optimized for the collection of each image, Z-stack steps were collected using Nyquist, 600 Hz, 1K x 1K, and 2.0 zoom. FITC fluorescence was determined in ImageJ using the time series analyzer plug in, deleting the out of focus slices for each 3D stack, and selecting the ROI using ImageJ’s freehand tool. Fluorescence was calculated for each slice and summed together to obtain the total fluorescence of each cell in 3D.

### Statistical analyses and software

Statistical analysis was carried out using GraphPad Prism 5 (GraphPad Software; San Diego, CA); student’s paired t-test was done to determine statistical significance for each assay as described in the figure legends. Microscopy image processing and analysis was done using ImageJ 1.50e (public domain) and Adobe Photoshop CS4 (Adobe Systems, Inc.; San Jose, CA).

## Results

### Evidence that JJX12 promotes the formation of multivalent ricin-antibody complexes on cell surfaces

In pilot studies, we observed that ricin was notably resistant to a competitive lactose wash when bound to HeLa cells in the presence of JJX12, as compared to ricin alone (S1 Fig). Specifically, ^125^I-ricin was mixed with JJX12 or JNA6 (Table 1) and then applied to HeLa cells at 4°C. Cells were then washed with 0.1 M lactose to competitively dissociate ricin from cell surfaces, after which the cells were lysed and the amount of cell-associated ricin was measured using a gamma counter. In the absence of antibody, only ~2.5% of the surface bound ricin was resistant to lactose. In the presence of JNA6, 10% surface bound ricin was resistant to lactose and in the presence of JJX12 that number increased to ~20%. These results were intriguing in light of the fact that JJX12 promotes the formation of higher order toxin-antibody complexes in solution, while JNA6 associated with ricin in 1:1 and 2:1 ricin:antibody complexes [25, 26]. We reasoned that the resistance of ricin-JJX12 to dissociation from cell surfaces in the presence of lactose could be due to increased valency (avidity) of polyvalent toxin-aggregates on cell surfaces [31].

**Fig 1:**
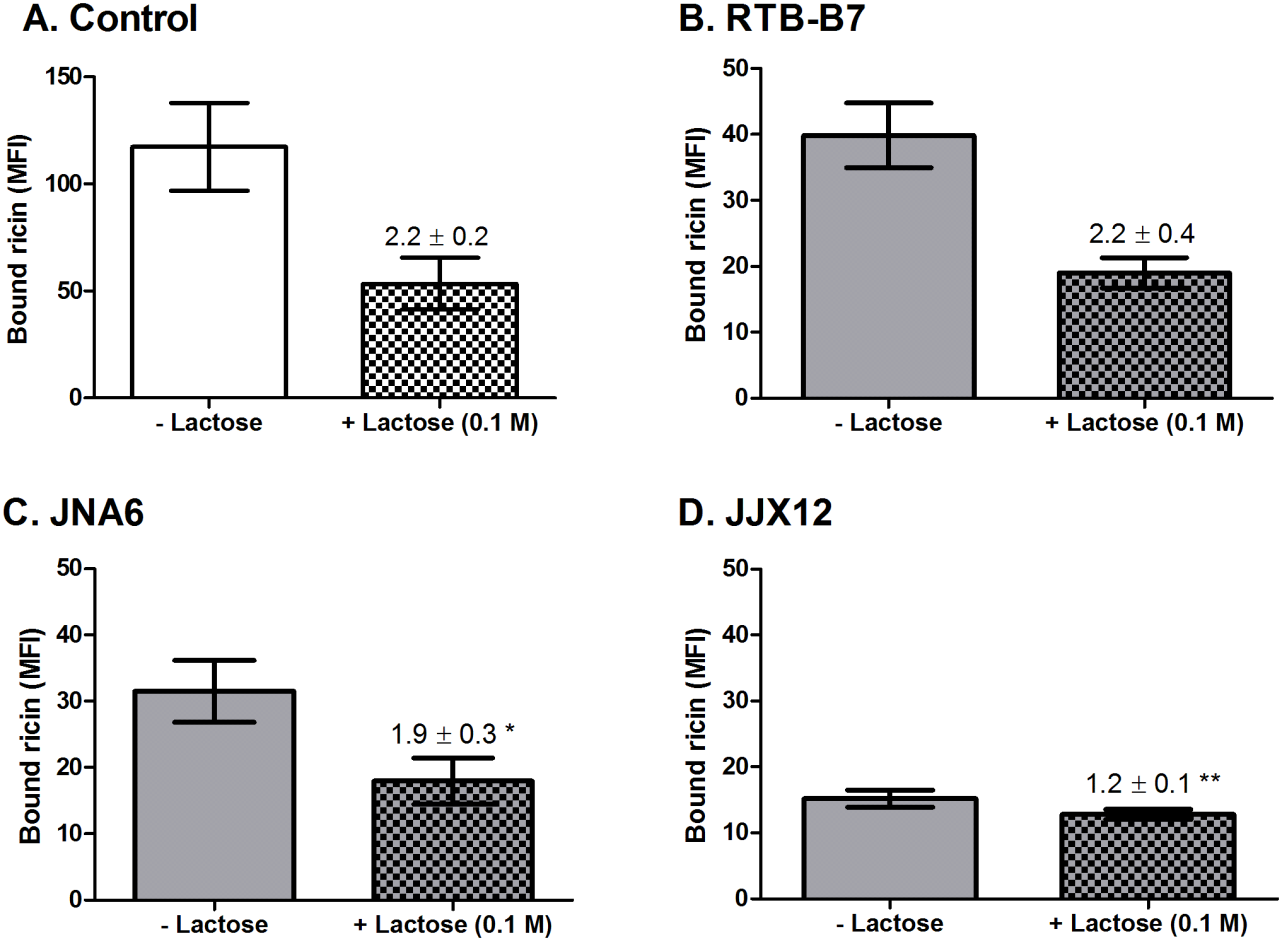
JJX12 reduces dissociation of ricin from THP-1 cell surfaces. THP-1 cells on ice were treated with (A) ricin-FITC or ricin-FITC with (B) RTB-B7, (C) JNA6, or (D) JJX12. Cells were then washed without or with 0.1 M lactose and subjected to flow cytometry, as described in Material and Methods. The amount of ricin-FITC bound to THP-1 cells on the y-axis is expressed as mean fluorescent intensity (MFI). Experiments were repeated three independent times and the bar graphs represent each treatment mean ± SD. The numbers over the hatched columns are the fold change ± SD in MFI (no lactose versus lactose treatment). Student’s paired t-test was done to determine statistical significance between fold change for ricin control and antibody treated cells (*p< 0.05 or **p< 0.01).

We employed a quantitative flow cytometry-based assay to test whether JJX12 affects the dissociation of ricin from cell surfaces. THP-1 cells (at 4°C) were treated with ricin-FITC in the absence or presence of JJX12, JNA6 or the monomer, RTB-B7, for 30 min, and then washed with 0.1 M lactose before being analyzed by flow cytometry for mean fluorescence intensity (MFI) (S1 Fig). Because the absolute amount of ricin bound to the cells in the presence of JJX12, JNA6, and RTB-B7 was different as it has been previously shown the antibodies affect ricin attachment to cell surfaces to varying degrees [26], the results of these experiments were expressed as the fold change in MFI (fold change = MFI without lactose/MFI 0.1 M lactose treatment) (Fig 1). In the case of ricin treated cells (without addition of antibody), a wash with 0.1 M lactose resulted in ≥ 2-fold reduction in MFI, indicating that more than half the surface bound ricin was removed (Fig 1). This reduction is less than what is routinely observed with HeLa cells, but similar to what we reported previously with this specific cell type [6, 32]. The addition of RTB-B7 did not affect ricin dissociation from THP-1 cells as compared to ricin controls (2.2 versus 2.2 MFI), although both JNA6 and JJX12 did. In the case of JNA6, ricin dissociation was marginally (although significantly) reduced, consistent with JNA6 partially stabilizing ricin on the cell surface through the formation of 2:1 ricin:antibody complexes (Fig 1). JJX12 had a much greater impact on ricin stabilization, as evidenced by only a 1.2-fold change in MFI following a lactose wash (Fig 1; S1 Fig). Using flow cytometry and Alexa647-labeled JJX12, we verified that JJX12 was indeed bound to ricin on the THP-1 cell surfaces in these experiments (S1 Fig).

We next examined the effect of JJX12 on ricin stability using a quantitative solid phase binding assay in which microtiter plates were coated with the glycoprotein ASF, probed with biotinylated ricin, without or with the co-administration of antibodies, then washed with lactose and probed with avidin-HRP. In this assay, dissociation of ricin from plate bound ASF was dose-dependent (Table 2; S2 Fig). As predicted, in the presence of JJX12, the dissociation of ricin from ASF by soluble lactose was significantly reduced (Table 2; S2 Fig). When treated with 0.4 mM lactose, 38% of ricin dissociated from ASF, whereas only 3% dissociated in the presence of JJX12. High lactose (10 mM) treatment resulted in 60% of ricin detachment from the plate, whereas only 21% detached when JJX12 was present. We verified by an ELISA that JJX12 remained bound to ricin following a lactose wash (S2 Fig).

**Table 2:** JJX12 affects ricin dissociation from a plate bound surrogate receptor.

	% Dissociation ^a^				JJX12 ^b^	
Lactose (mM)	Ricin	+RTB-B7	+JNA6	+JJX12	vs. RTB-B7	vs. JNA6
0.4	37.7 ± 5.6	22.8 ± 4.8	7.6 ± 1.0	3.0 ± 1.8	*p<* 0.001	*ns*
1	52.9 ± 3.7	38.6 ± 4.6	21.7 ± 0.65	10.0 ± 2.6	*p<* 0.05	*p<* 0.05
10	61.7 ± 1.2	42.7 ± 9.2	41.8 ± 1.7	21.3 ± 3.6	*ns*	*p<* 0.001

^*a*,^ Dissociation shown in percentages ± SD;

^b,^ Student paired t-test comparing ricin to ricin plus JJX12, JNA6, or RTB-B7.

*ns*, not significant.

**Fig 2:**
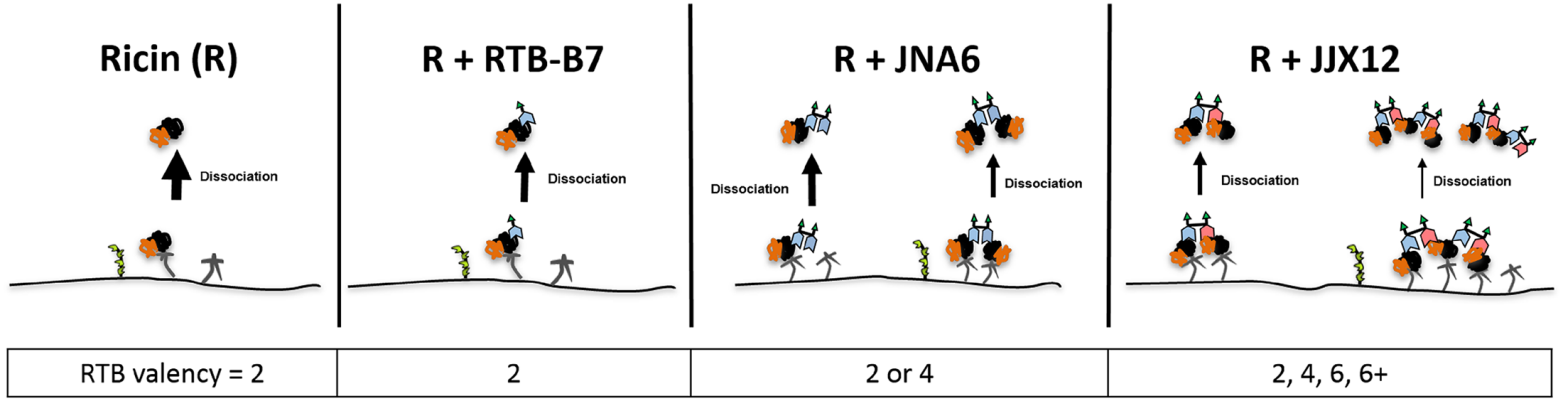
Proposed model to explain how JJX12 decreases ricin dissociation from cell surfaces. Ricin holotoxin consisting of RTA (orange) and RTB (black) binds to Gal/GalNAc receptors (gray) and/or the mannose receptor (green) on the surface of mammalian cells. RTB-specific (blue) and RTA-specific (pink) V_H_Hs are shown as monomers, dimers or heterdimers. As shown in the left panel, ricin has low affinity (high dissociation; bold arrow) for terminal Gal/GalNac containing glycoproteins/glycolipids on the cell surface [6]. RTB has two carbohydrate binding sites, corresponding to a valency of 2. The addition of monomeric (RTB-B7), homodimeric (JNA6), or bispecific (JJX12) V_H_Hs are expected to reduce dissociation of ricin because of corresponding changes in valency.

To investigate to what degree JJX12’s effects on ricin’s dissociation were attributable to crosslinking, we repeated the above experiments in the presence of bivalent monospecific antibody JNA6, or monovalent antibody RTB-B7 (Tables 1 and 2). Tabulation of the results demonstrate that dissociation of ricin following a lactose wash was significantly less when bound to JJX12, as compared to either JNA6 or RTB-B7 (Table 2). The differences between JJX12 and JNA6 were only evident at the higher lactose concentrations, an observation that is consistent with JNA6 stabilizing RTB dimers on ASF that are resistant to low lactose concentrations, whereas JJX12 forms higher order RTB-antibody complexes resistant to high lactose concentrations (Fig 2).

#### Ricin:JJX12 aggregates are endocytosed via a macropinocytosis-like mechanism

We estimate that the size distribution of ricin-JJX12 complexes (in solution) is between 22 and 50 nm [25, 26], which would be expected to have consequences in terms of how the toxin is internalized and trafficked by HeLa and A549 cells. Whereas ricin uptake occurs through receptor-mediated clathrin-dependent and independent endocytosis, ~30 nm diameter quantum dots coated with RTB (RTB-QDs) are internalized through a dynamin-dependent macropinocytosis-like mechanism in HeLa cells [33]. Macropinocytosis is an actin- and dynamin-dependent uptake process that is amiloride-sensitive (Na+/H+ exchanger in plasma membrane). Distinct steps in macropinocytosis can be probed using three different inhibitors: the amiloride analog EIPA, which effects Na+/H+ exchange at the plasma membrane [34]; LatA, which depolymerizes F-actin [35]; and dynasore, which inhibits dynamin from pinching off endocytic vesicles [36].

To examine a role for macropinocytosis in the uptake of ricin-JJX12 complexes, the adherent human lung epithelial A549 cells were treated with ricin-FITC in the absence or presence of JJX12 for 30 min at 37°C, fixed and imaged by confocal microscopy, as described in the Materials and Methods. To correct for effects that JJX12 and JNA6 may have on the total amount of ricin that gains entry into cells, all comparisons (and statistical analyses) were done between without and with inhibitor for each antibody.

**Fig 3:**
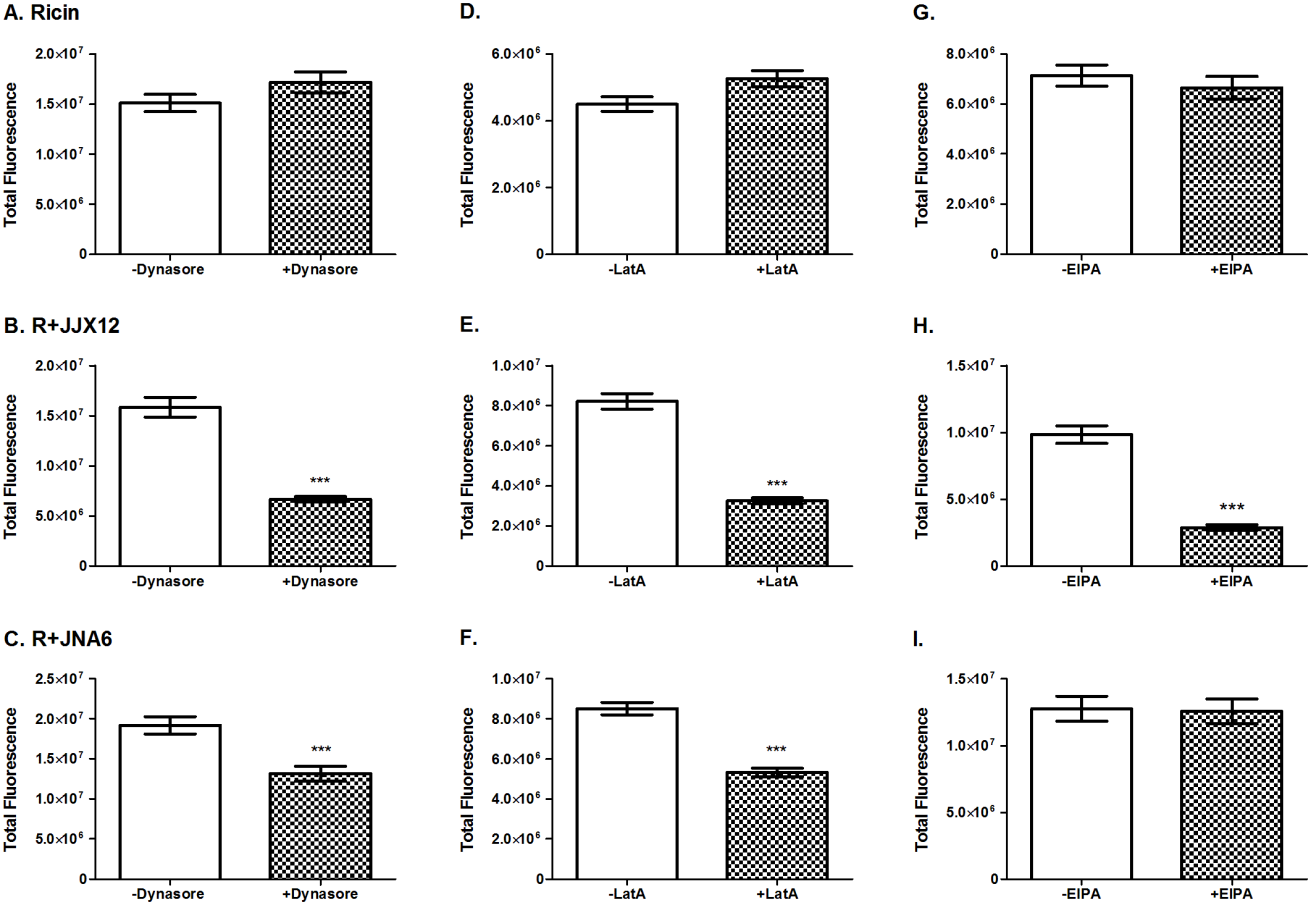
Ricin-JJX12 complexes are endocytosed via a macropinocytosis-like mechanism. A549 cells were treated with (panels A-C) 100 μM dynasore, (panels D-F) 0.6 μM LatA or (panels G-I) 200 μM EIPA, as described in the Materials and Methods. The cells were then treated with ricin-FITC, ricin-FITC with JJX12 or ricin-FITC with JNA6. Cells were then washed, fixed, and analyzed to determine the total amount of ricin-FITC internalization untreated (white bars) and treated cells (checked bars). N = 40 cells; bar graphs represent each treatment mean ± SEM. Student’s paired t-test was done to determine statistical significance between untreated controls and treated cells (***p< 0.001).

In the absence of JJX12, ricin-FITC uptake into A549 cells resulted in localization of the toxin within small vesicular compartments distributed throughout the cell. A fraction of ricin-FITC remained bound to the cell membrane at the 30 min time point, indicating that ricin uptake is asynchronous [27, 37]. Treatment of A549 cells with EIPA, LatA, or dynasore did not affect the total amount of ricin-FITC that was internalized (Fig 3; S3–S5 Figs). On the other hand, ricin uptake in the presence of JJX12 was significantly reduced when cells were treated with dynasore (58%; *p* < 0.001), LatA (60%; *p*< 0.001), and EIPA (70%; *p* < 0.001), consistent with uptake of ricin-JJX12 complexes via a macropinocytosis-like mechanism. We confirmed using Alexa633-labeled JJX12 that ricin was invariably co-localized with JJX12 on cell surfaces and internalized as an antibody complex (**data not shown**).

By way of comparison, we repeated the studies with JNA6. Based on AUC, we estimate that JNA6-ricin complexes range in size between 14 nm and 21 nm in diameter [26]. In the presence of JNA6, ricin uptake was reduced when A549 cells were treated with dynasore (31.5%; *p* < 0.001) and LatA (37.5%; *p* < 0.001), but not EIPA (Fig 3; S3–S5 Figs). These results suggest that JNA6 is endocytosed via a dynamin- and actin-dependent mechanism, but likely not macropinocytosis. As before, we confirmed by direct labeling of JNA6 with Alexa633 that ricin-antibody complexes were internalized as an ensemble (**data not shown**).

#### JJX12 affects retrograde transport of ricin toxin to the TGN

**Fig 4:**
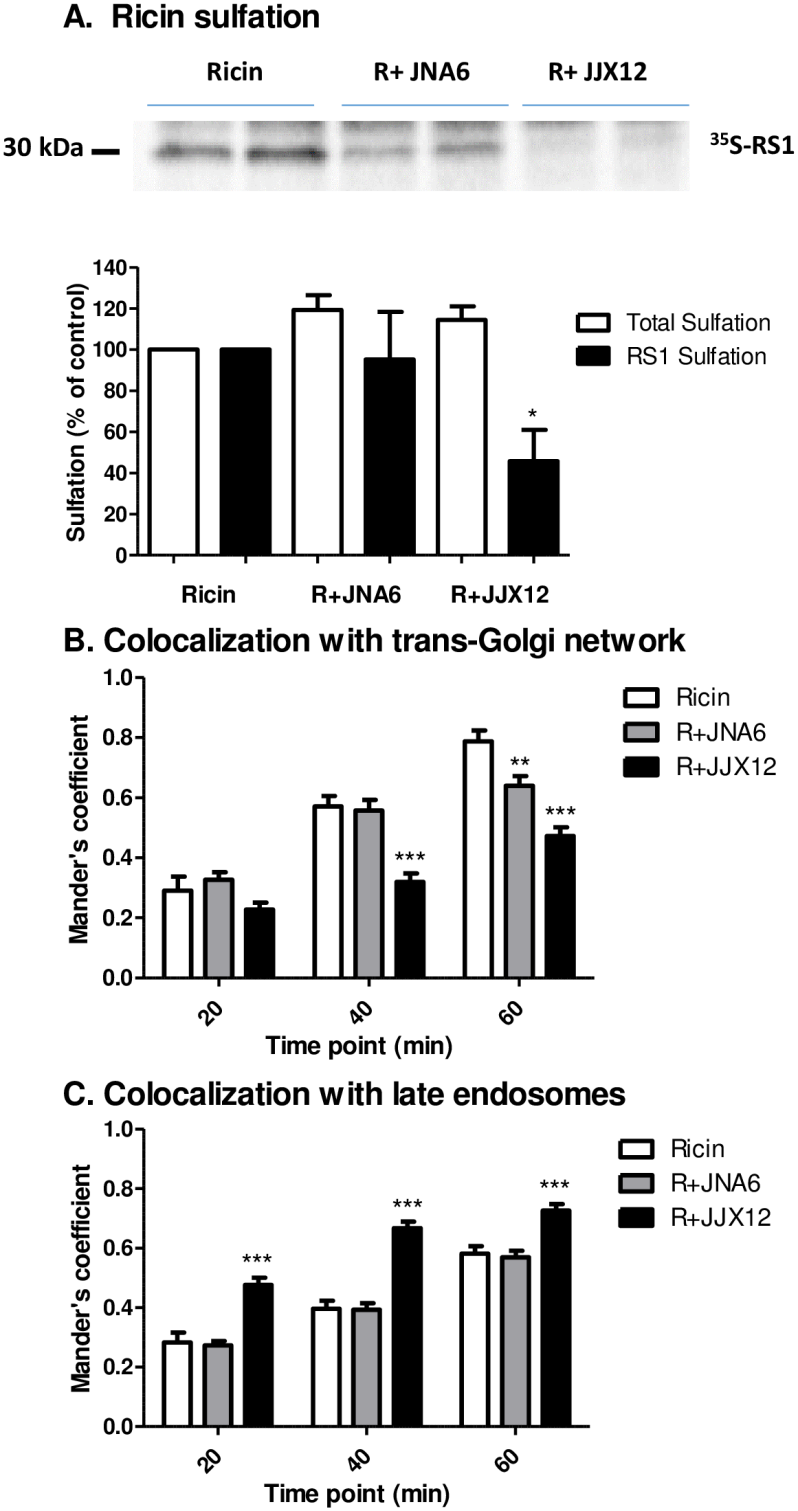
JJX12 effects retrograde transport of ricin. (Panel A) For ricin sulfation studies, HeLa cells were incubated with Na_2_^35^SO_4_ before incubation of RS1 (10 μg) alone or mixed with antibody (10 μg) for 2 h. Cells were washed twice with lactose to remove unbound and cell surface-associated ricin. Specific RTA sulfation was measured by autoradiography (top panel) and quantitated by densitometry (bottom panel; black bars). Total sulfation was determined by quantitating precipitation of the remaining lysate (white bars), as described in the Materials and Methods. Experiments were done three independent times and the bar graphs represent mean ± SD. Student paired t-test was done to determine statistical significance for RS1 sulfation between ricin control and antibody treated cells (*p< 0.05). (Panels B-C) For co-localization studies, A549 cells were transiently transfected with RFP-tagged organelle specific proteins to label the (panel B) TGN or (panel C) late endosomes, as described in Materials and Methods. After transfection, cells were incubated with ricin-FITC (white bar) or ricin-FITC mixed with JNA6 or JJX12 (black bar) for 20 min at 37°C. Cells were washed to remove unbound ricin or ricin-antibody complexes and imaged in real time for 60 min. Mander’s coefficient values were determined to quantitate co-localization at 20, 40, and 60 min. At least 20 cells were quantitated for each time point per treatment; bar graphs represent the mean ± SEM. Student paired t-test was done to determine statistical significance between ricin controls and antibody treatment (**p< 0.01 or ***p< 0.001).

There is evidence that size affects the efficiency by which particles are internalized and transported retrogradely to the TGN [33, 38–41]. Based on these reports, we postulated that transport of ricin to the TGN would be affected by JJX12. Using a combination of biochemical and live cell imaging techniques, we first compared the efficiency of ricin toxin transport to the TGN in the absence and presence of JJX12 or JNA6. Trafficking of ricin to the TGN was measured using a well established organelle-specific reporter system in which a derivative of RTA (“RS1”) becomes modified by resident tyrosylprotein sulfotransferase upon entry into the TGN [28]. Using this assay, we found that RS1 sulfation decreased by 54% (*p* < 0.05) in the presence of JJX12, as compared to RS1 alone (Fig 4A). JNA6, on the other hand, had no significant effect on RS1 sulfation, demonstrating that simple association of an antibody with ricin is not itself sufficient to alter retrograde transport to the TGN (Fig 4).

We next used live cell imaging to determine the fate of ricin-JJX12 complexes following endocytosis in A549 cells. To tag the TGN, A549 cells were transiently transfected with a construct that expresses RFP fused to the human Golgi resident enzyme N-acetylgalactosaminyltransferase, as described in the Materials and Methods. The transfected cells were then pulsed with ricin-FITC, in the absence and presence of JJX12 and JNA6. A549 cells were imaged in real time and co-localization of ricin-FITC with the TGN was quantitated at 20, 40, and 60 min time points (Fig 4B; S1–S3 Movies). In the presence of JJX12, ricin accumulation in the TGN was significantly reduced as compared to the ricin control at 40 and 60 min (Fig 4B; S1–S3 Movies). Ricin-JNA6 co-localization with the TGN was unaltered except for a slight reduction at the 60 min time point. Thus, these results are in accordance with the results obtained by RS1 sulfation assays.

Finally, we investigated whether ricin-JJX12 complexes accumulate in the late endosome and/or lysosomes, using RFP-Rab7a and RFP-Lamp1, respectively. Live cell imaging of transfected cells demonstrated that ricin-JJX12 aggregates accumulated in late endosomes as demonstrated by co-localization with RFP-Rab7a (Fig 4C; S4–S6 Movies). However, at the time points examined (30, 60 and 90 min) there was no preferential co-localization of ricin-JJX12 complexes in lysosomes (S6 Fig). JNA6 has no effect on ricin retrograde transport based on the observation that ricin-JNA6 complexes had co-localization profiles that were identical to ricin (Fig 4B and 4C; S6 Fig; S1–S6 Movies). These results are consistent with JJX12 affecting ricin retrograde trafficking to the TGN.

## Discussion

In this study, we present evidence that JJX12 promotes the formation of ricin-antibody aggregates on the cell surface, which affects toxin uptake and trafficking to the TGN. Using THP-1 and HeLa cells, we demonstrated that in the presence of JJX12, a large fraction of cell-bound ricin was resistant to dissociation upon the addition of 0.1 M lactose. Similar results were observed in a cell-free microtiter plate-based assay using ASF as a surrogate receptor. Based on known kinetic parameters of ricin association with, and dissociation from cell surfaces, we interpret ricin’s resistance to lactose competition in the presence of JJX12 as being indicative of effects on valency (Fig 2). Specifically, it is estimated that there are ~1x10^7^ ricin binding sites per HeLa cell [6]. The binding of ricin to Gal/GalNAc moieties on surface exposed glycoproteins and glycolipids is mediated by RTB’s two carbohydrate recognition domains (CRDs), which are situated on opposite poles of RTB and thought to function independently (*i*.*e*., no evidence of cooperatively) [5, 6, 42]. Thus, under conditions where cells are maintained at 4°C to prevent endocytosis, changes in ricin dissociation from cell surfaces upon excess lactose exposure are most likely due to changes in RTB avidity (Fig 2). For example, it has been shown using MCF-7 and Vero cells and electron microscopy that monovalent ricin-HRP was easily removed from the cells surface with 0.1 M lactose, while polyvalent ricin-HRP or polyvalent ricin-gold conjugates are not [27]. We propose that JJX12 can concatenate ricin in solution or on cell surfaces, as evidenced by JJX12’s effects on ricin dissociation whether the antibody was mixed with toxin in solution or after ricin had bound to cells or ASF-coated microtiter plates.

We further provide evidence, through the use of confocal microscopy and live cell imaging, that JJX12 affects the dynamics of ricin uptake and intracellular transport. Uptake of ricin in the presence of JJX12 was significantly reduced when cells were treated with dynasore, LatA, and EIPA, a profile that is consistent with internalization occurring via a macropinocytosis-like mechanism. JJX12 also reduced retrograde transport of ricin to the TGN and correspondingly enhanced toxin accumulation in late endosomes. These results are consistent with several findings from the literature that demonstrate that size and valency influence ricin uptake and intracellular transport. For example, it was recently reported that simply conjugating RTB to 30 nm in diameter quantum dots (RTB-QD) results in toxin uptake into HeLa cells via a dynamin-dependent macropinocytosis-like mechanism, similar to what we observed with JJX12 [33]. It was also shown decades ago by transmission electron microscopy that monovalent ricin-HRP is successfully trafficked to TGN within 45 min, whereas polyvalent ricin-HRP fails to reach that same compartment [27]. Thus, there is substantial evidence that intracellular routing of ricin is affected by multimerization or aggregation, which in turn would be expected to result in localized receptor clusters. In the case of Shiga and cholera toxins, clustering of surface glycosphingolipids (GSLs) has important consequences for intracellular transport [43]. It is interesting to note that JNA6 neutralizes ricin *in vitro*, but does so without interfering with ricin trafficking to the TGN. JNA6 must, therefore, neutralize ricin by interfering with egress from TGN [44], transport to the ER [28], and/or retro-translocation [45].

**Fig 5:**
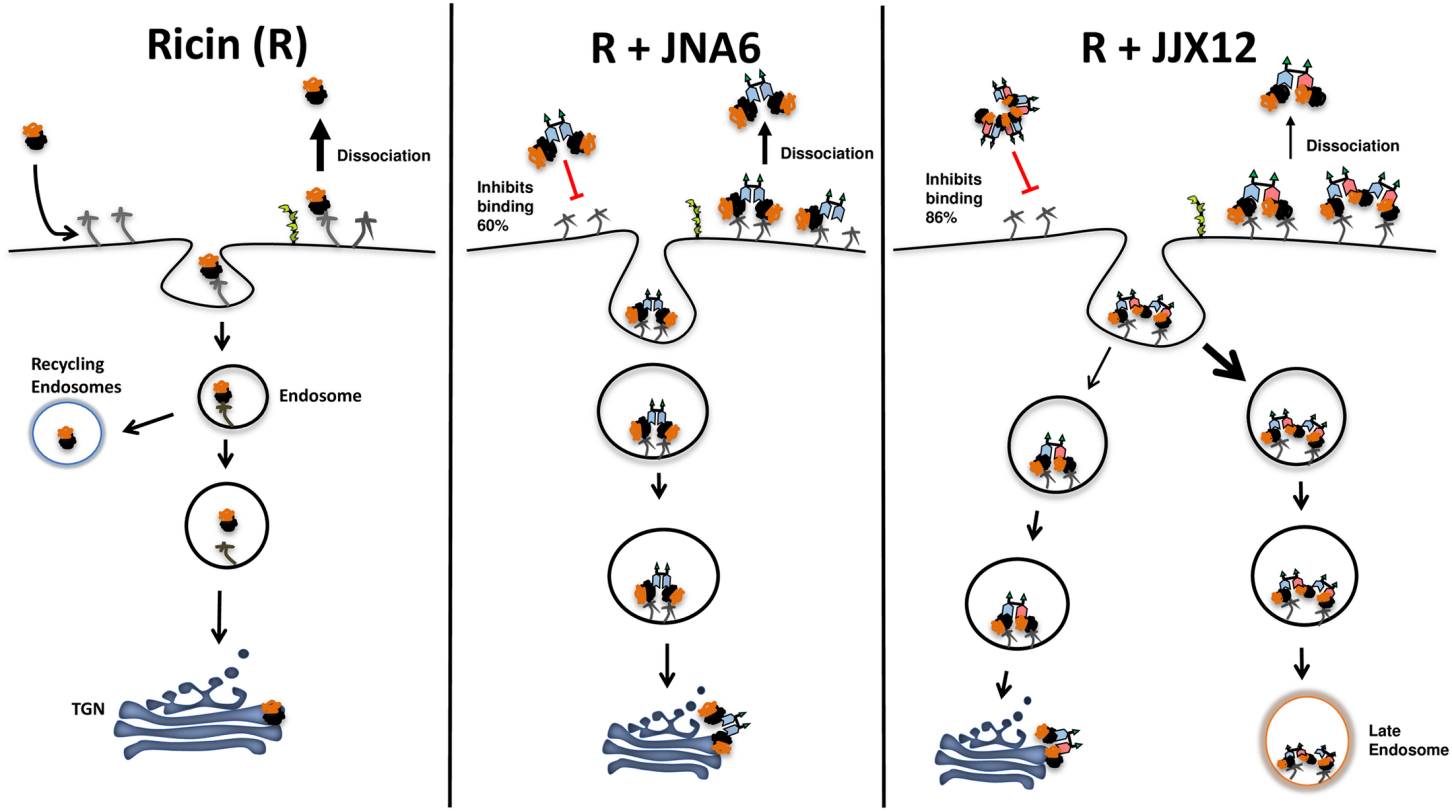
Proposed effects of JJX12 on ricin dissociation and internalization. (Left) Uptake and trafficking of ricin, which has low affinity for cell surface receptors [6]. (Middle) JNA6 partially inhibits ricin from binding to cell surfaces [26] but had no effect on ricin retrograde transport. (Right) JJX12 interferes with ricin binding to cells surfaces, reduces ricin dissociation from membrane receptors, and promotes uptake via a macropinocytosis-like mechanism, which results in reduced transport to the TGN and enrichment of ricin in late endosomes.

In summary, we propose that JJX12 has effects on ricin toxin in extracellular and intracellular compartments (Fig 5). In terms of the extracellular space, we previously demonstrated that JJX12 reduces ricin binding to cell surfaces, presumably because RTB’s access to host cells receptors is occluded in ricin-antibody aggregates [25, 26]. In terms of the intracellular space, we now report that JJX12 decreases ricin trafficking to the TGN, presumably because ricin-antibody complexes present on cell surfaces are shunted to the late endosomes for degradation. Thus, we speculate that JJX12’s potent neutralizing *in vivo* activity is likely due to serial triage (*i*.*e*., interference with multiple steps in the cytotoxic pathway). In this sense, JJX12 may mimic effects normally associated with oligoclonal mixtures of antibodies. Whether JJX12’s activities are shared with other engineered bispecific antibodies such as those against BoNT [14], *Clostridium difficile* toxins TcdA and TcdB [15–17], Shiga toxins [18], and anthrax toxin [19] remains to be determined. The ongoing efforts to study VHHs and understand their neutralizing mechanisms have opened up new avenues for the rational design and delivery of antitoxin agents [13].

## Supporting Information

S1 Fig JJX12 decreases ricin’s ability to dissociate from cell surfaces. (A) To quantitate lactose-resistance of ricin bound to HeLa cells, cells were first treated with ^125^I-ricin (50 ng/ml) mixed with JNA6 or JJX12 (0.5 μg) and incubated for 30 min on ice to prevent endocytosis of complexes. Cells were then washed with PBS or 0.1 M lactose, and lysed. Cell-associated ^125^I-ricin was quantitated and presented as percent cell associated ricin after lactose compared to PBS wash for each condition. Experiments were performed twice with triplicate samples, giving similar results. (B) Representative FACS plot of THP-1 cells treated with ricin-FITC, JJX12-Alexa647, or ricin-FITC mixed with JJX12-Alexa647, shows positive cells for FITC on the x-axis and positive for Alexa647 on the y-axis.
(TIF)

S2 Fig Effect on ricin’s dissociation from ASF in the presence of lactose. For lactose competition ELISAs, plates were coated with ASF overnight and then probed with ricin (3 μg/mL) mixed with RTB-B7 (24.4 μg/mL), JNA6 or JJX12 (18.5 μg/mL) for 1 h. Plates where then incubated with two-fold serial dilution of lactose starting at 0.2 M for 1 h. (A) Plate bound ricin was detected using avidin-HRP and optical density (OD) was measured at 450 nm and shown on the y-axis. (B) VHHs bound to ricin were detected using anti-E-tag HRP as described in Material and Methods. The results represent a single representative experiment done in triplicate with each data point indicating mean ± SD. In some instances, error bars are masked by symbol and therefore not visible.
(TIF)

S3 Fig Effects on ricin uptake in dynasore treated cells. Cells were treated with dynasore (100 μM) for 30 min at 37°C. Ricin-FITC (15 μg/mL) was incubated with JJX12 or JNA6 (101.64 μg/mL) for 15 min prior to adding to A549 cells. Cells were incubated with ricin alone or mixture for 20 min at 37°C, washed to remove unbound ricin, and incubated for 30 min with only inhibitors. Cells were fixed as described in Materials and Methods and imaged using confocal microscopy. Representative images of cells treated without (left panel) or with (right panel) chemical inhibitor. Red trace outlines a representative cell, and white arrows highlight examples of ricin-FITC (gray shading) inside cells. Scale bar 20 μm.
(TIF)

S4 Fig Effects on ricin uptake in LatA treated cells. Cells were treated with LatA (0.6 μM) for 30 min at 37°C. Ricin-FITC incubated with JJX12 or JNA6 for 15 min prior to adding to A549 cells. Cells were incubated with ricin alone or mixture for 20 min at 37°C, washed to remove unbound ricin, and incubated for 30 min with only inhibitors. Cells were fixed and imaged using confocal microscopy. Representative images of cells treated without (left panel) or with (right panel) chemical inhibitor. Red trace outlines a representative cell, and white arrows highlight examples of ricin-FITC (gray shading) inside cells. Scale bar 20 μm.
(TIF)

S5 Fig Effects on ricin uptake in EIPA treated cells. Cells were treated with EIPA (200 μM) for 30 min at 37°C. Cells were incubated with ricin alone or mixture for 20 min at 37°C, then washed to remove unbound ricin, and incubated for 30 min with only inhibitors. Cells were fixed and imaged using confocal microscopy. Representative images of cells treated without (left panel) or with (right panel) chemical inhibitor. Red trace outlines a representative cell, and white arrows highlight examples of ricin-FITC (gray shading) inside cells. Scale bar 20 μm.
(TIF)

S6 Fig JJX12-ricin complexes accumulate in the lysosomes. For co-localization studies, A549 cells were transfected to label the lysosomes as described in Material and Methods. After transfection, cells were incubated with ricin-FITC (white bar) or ricin-FITC mixed with JNA6 (gray bar) or JJX12 (black bar) for 20 min at 37°C, washed to remove unbound ricin, and incubated for an additional 30, 60 or 90 min prior to fixing and imaging using confocal microscopy. Mander’s coefficient values to quantitate co-localization of ricin and lysosomes was determined for each time point. At least 20 cells were quantitated for each time point and treatment; bar graphs represent the mean ± SEM.
(TIF)

S1 Movie Live cell imaging of ricin-FITC and TGN. Real time imaging of A549 cells visualizing ricin-FITC in green and TGN in red labeled with a transient transfection as described in Material and Methods. The total fluorescence for the TGN marker is always overwhelming compared to ricin-FITC and therefore the absolute co-localization between ricin-FITC and the TGN is not apparent in the raw images prior to processing. Real time images were processed prior to quantification as described in Materials and Methods to determine co-localization values, which are reported in Fig 4B. Total length of the time lapse is 120 min.
(AVI)

S2 Movie Live cell imaging of ricin-FITC in the presence of JNA6 and TGN. Real time imaging of A549 cells visualizing ricin-FITC in green pre-mixed with JNA6 prior to adding to cells and TGN in red labeled with a transient transfection as described in Material and Methods. The total fluorescence for the TGN marker is always overwhelming compared to ricin-FITC and therefore the absolute co-localization between ricin-FITC and the TGN is not apparent in the raw images prior to processing. Real time images were processed prior to quantification as described in Materials and Methods to determine co-localization values, which are reported in Fig 4B. Total length of the time lapse is 120 min.
(AVI)

S3 Movie Live cell imaging of ricin-FITC in the presence of JJX12 and TGN. Real time imaging of A549 cells visualizing ricin-FITC in green pre-mixed with JJX12 prior to adding to cells and TGN in red labeled with a transient transfection as described in Material and Methods. The total fluorescence for the TGN marker is always overwhelming compared to ricin-FITC and therefore the absolute co-localization between ricin-FITC and the TGN is not apparent in the raw images prior to processing. Real time images were processed prior to quantification as described in Materials and Methods to determine co-localization values, which are reported in Fig 4B. Total length of the time lapse is 120 min.
(AVI)

S4 Movie Live cell imaging of ricin-FITC and late endosomes. Real time imaging of A549 cells visualizing ricin-FITC in green and late endosomes in red labeled with a transient transfection as described in Material and Methods. The total fluorescence for the late endosomes marker is always overwhelming compared to ricin-FITC and therefore the absolute co-localization between ricin-FITC and the late endosomes is not apparent in the raw images prior to processing. Real time images were processed prior to quantification as described in Materials and Methods to determine co-localization values, which are reported in Fig 4C. Total length of the time lapse is 90 min.
(AVI)

S5 Movie Live cell imaging of ricin-FITC in the presence of JNA6 and late endosomes. Real time imaging of A549 cells visualizing ricin-FITC in green pre-mixed with JNA6 prior to adding to cells and late endosomes in red labeled with a transient transfection as described in Material and Methods. The total fluorescence for the late endosomes marker is always overwhelming compared to ricin-FITC and therefore the absolute co-localization between ricin-FITC and the late endosomes is not apparent in the raw images prior to processing. Real time images were processed prior to quantification as described in Materials and Methods to determine co-localization values, which are reported in Fig 4C. Total length of the time lapse is 90 min.
(AVI)

S6 Movie Live cell imaging of ricin-FITC in the presence of JJX12 and late endosomes. Real time imaging of A549 cells visualizing ricin-FITC in green pre-mixed with JJX12 prior to adding to cells and late endosomes in red labeled with a transient transfection as described in Material and Methods. The total fluorescence for the late endosomes marker is always overwhelming compared to ricin-FITC and therefore the absolute co-localization between ricin-FITC and the late endosomes is not apparent in the raw images prior to processing. Real time images were processed prior to quantification as described in Materials and Methods to determine co-localization values, which are reported in Fig 4C. Total length of the time lapse is 90 min.
(AVI)
