# Supplementary figures and images for: A Bispecific Antibody Promotes Aggregation of Ricin Toxin on Cell Surfaces and Alters Dynamics of Toxin Internalization and Trafficking
Source: PLoS One. 2016 Jun 14;11(6):e0156893. doi: 10.1371/journal.pone.0156893 (PMC4907443; doi:10.1371/journal.pone.0156893)

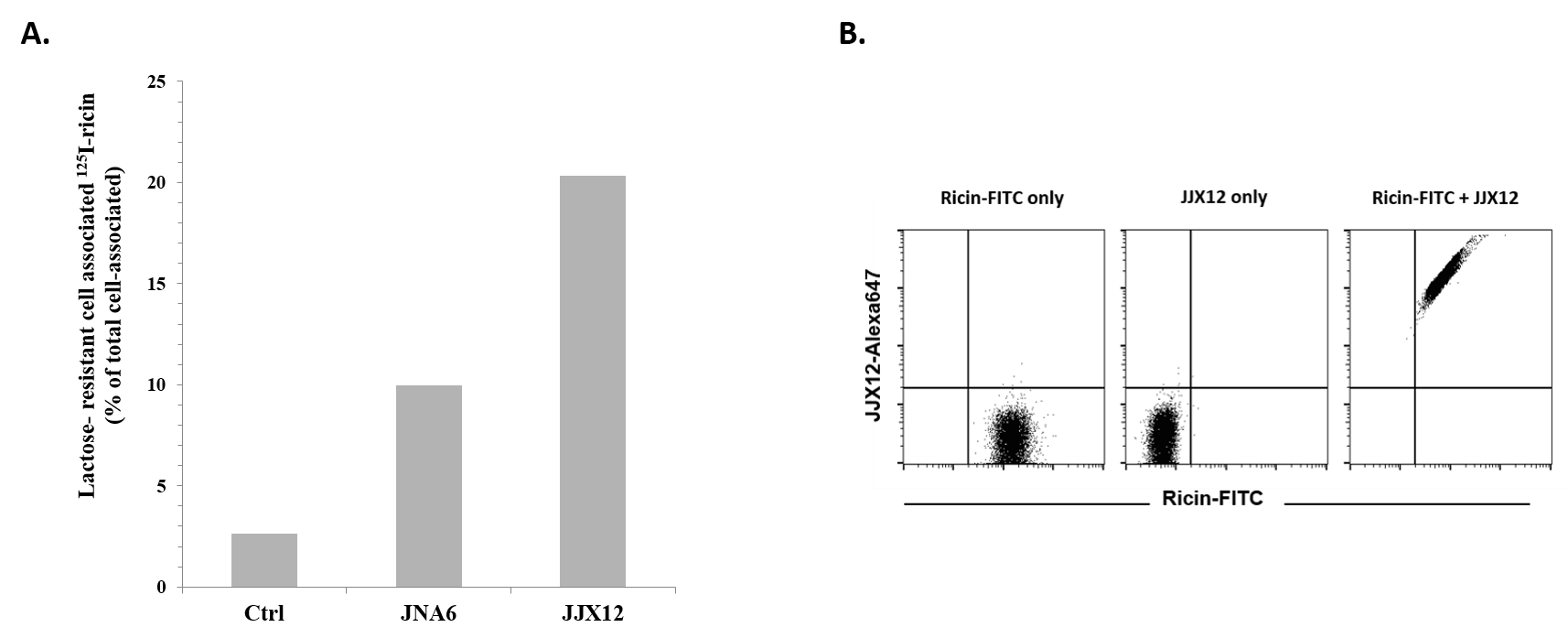

Supplement: S1 Fig — (A) To quantitate lactose-resistance of ricin bound to HeLa cells, cells were first treated with 125I-ricin (50 ng/ml) mixed with JNA6 or JJX12 (0.5 μg) and incubated for 30 min on ice to prevent endocytosis of complexes. Cells were then washed with PBS or 0.1 M lactose, and lysed. Cell-associated 125I-ricin was quantitated and presented as percent cell associated ricin after lactose compared to PBS wash for each condition. Experiments were performed twice with triplicate samples, giving similar results. (B) Representative FACS plot of THP-1 cells treated with ricin-FITC, JJX12-Alexa647, or ricin-FITC mixed with JJX12-Alexa647, shows positive cells for FITC on the x-axis and positive for Alexa647 on the y-axis. (TIF) [file pone.0156893.s001.tif]

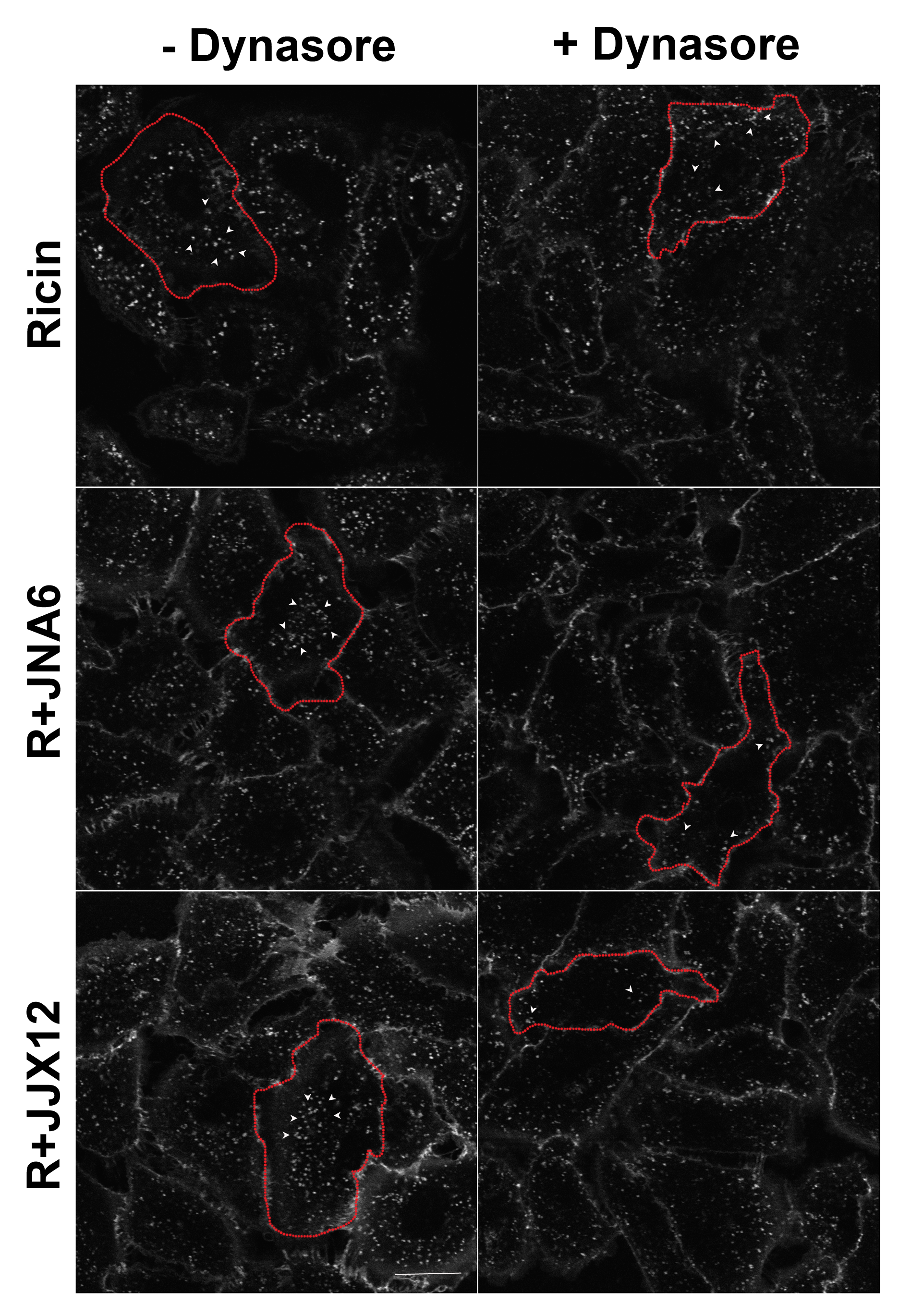

Supplement: S3 Fig — Cells were treated with dynasore (100 μM) for 30 min at 37°C. Ricin-FITC (15 μg/mL) was incubated with JJX12 or JNA6 (101.64 μg/mL) for 15 min prior to adding to A549 cells. Cells were incubated with ricin alone or mixture for 20 min at 37°C, washed to remove unbound ricin, and incubated for 30 min with only inhibitors. Cells were fixed as described in Materials and Methods and imaged using confocal microscopy. Representative images of cells treated without (left panel) or with (right panel) chemical inhibitor. Red trace outlines a representative cell, and white arrows highlight examples of ricin-FITC (gray shading) inside cells. Scale bar 20 μm. (TIF) [file pone.0156893.s003.tif]

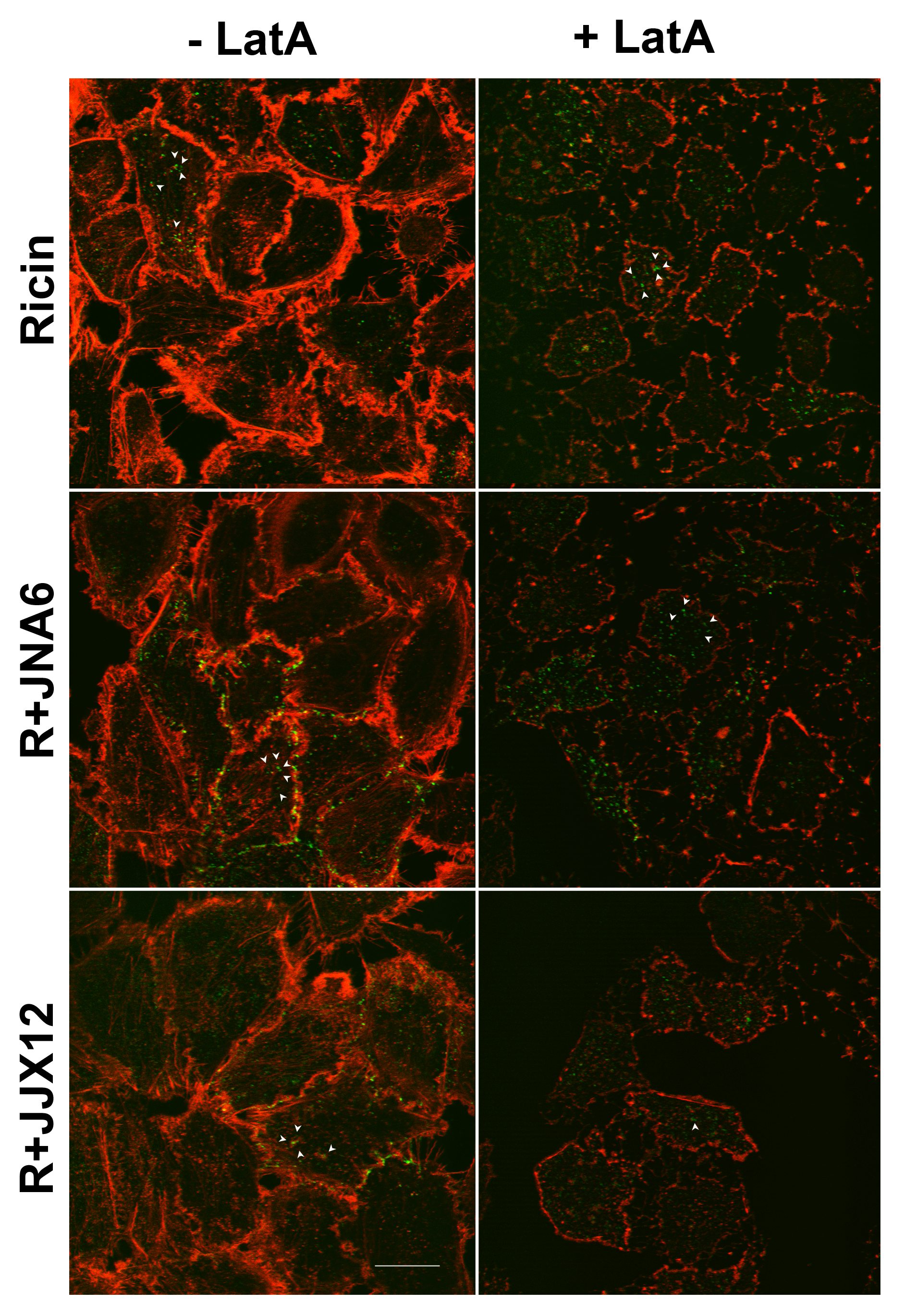

Supplement: S4 Fig — Cells were treated with LatA (0.6 μM) for 30 min at 37°C. Ricin-FITC incubated with JJX12 or JNA6 for 15 min prior to adding to A549 cells. Cells were incubated with ricin alone or mixture for 20 min at 37°C, washed to remove unbound ricin, and incubated for 30 min with only inhibitors. Cells were fixed and imaged using confocal microscopy. Representative images of cells treated without (left panel) or with (right panel) chemical inhibitor. Red trace outlines a representative cell, and white arrows highlight examples of ricin-FITC (gray shading) inside cells. Scale bar 20 μm. (TIF) [file pone.0156893.s004.tif]

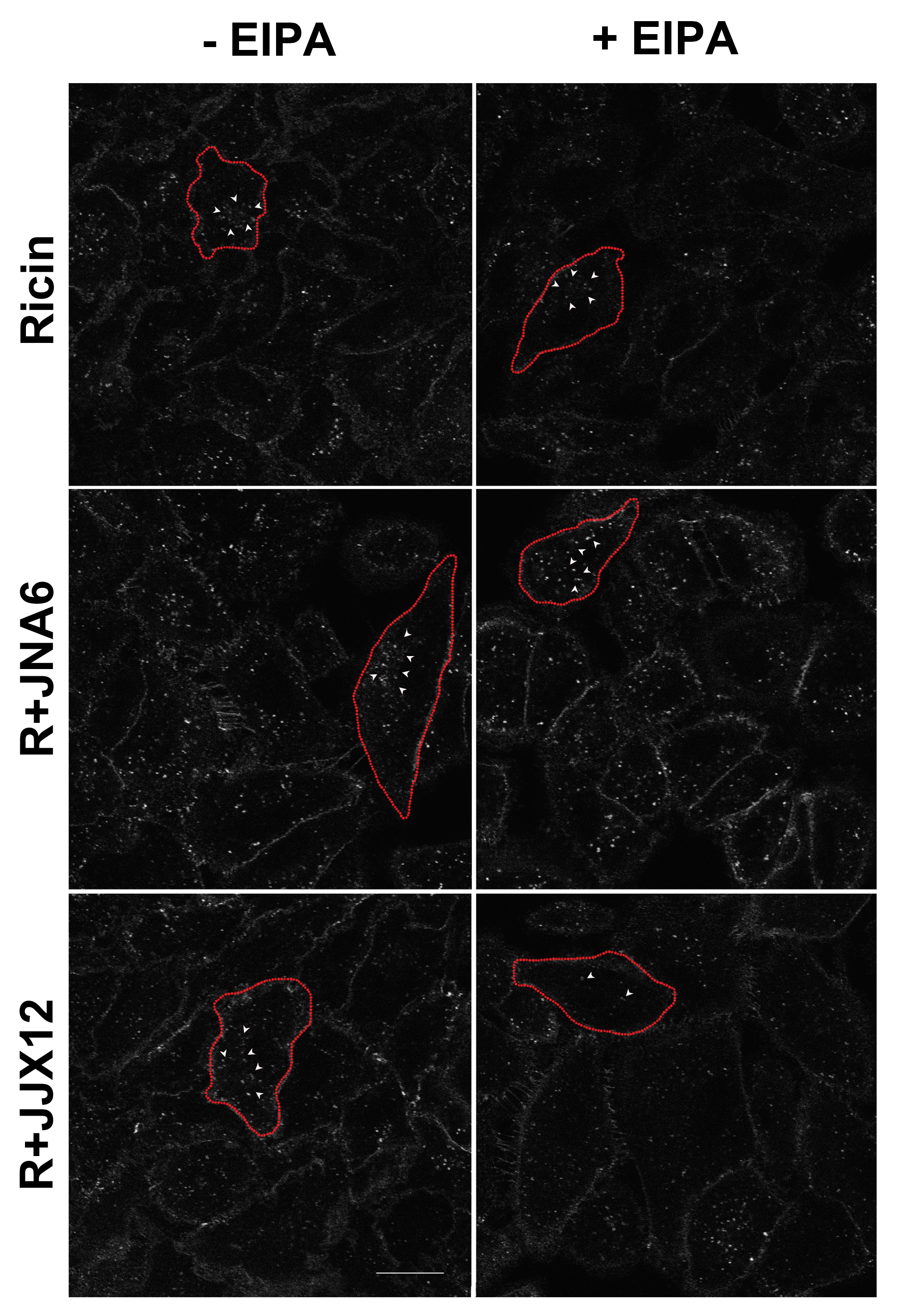

Supplement: S5 Fig — Cells were treated with EIPA (200 μM) for 30 min at 37°C. Cells were incubated with ricin alone or mixture for 20 min at 37°C, then washed to remove unbound ricin, and incubated for 30 min with only inhibitors. Cells were fixed and imaged using confocal microscopy. Representative images of cells treated without (left panel) or with (right panel) chemical inhibitor. Red trace outlines a representative cell, and white arrows highlight examples of ricin-FITC (gray shading) inside cells. Scale bar 20 μm. (TIF) [file pone.0156893.s005.tif]
